# Potency of umbilical cord blood- and Wharton’s jelly-derived mesenchymal stem cells for scarless wound healing

**DOI:** 10.1038/srep18844

**Published:** 2016-01-05

**Authors:** Hanako Doi, Yuriko Kitajima, Lan Luo, Chan Yan, Seiko Tateishi, Yusuke Ono, Yoshishige Urata, Shinji Goto, Ryoichi Mori, Hideaki Masuzaki, Isao Shimokawa, Akiyoshi Hirano, Tao-Sheng Li

**Affiliations:** 1 Department of Stem Cell Biology, Atomic Bomb Disease Institute, Nagasaki University, 1-12-4 Sakamoto, Nagasaki 852-8523, Japan; 2 Department of Plastic and Reconstructive Surgery, Nagasaki University Graduate School of Biomedical Sciences, 1-12-4 Sakamoto, Nagasaki 852-8523, Japan; 3 Department of Obstetrics and Gynecology, Nagasaki University Graduate School of Biomedical Sciences, 1-12-4 Sakamoto, Nagasaki 852-8523, Japan; 4 Department of Pathology, Nagasaki University Graduate School of Biomedical Sciences, 1-12-4 Sakamoto, Nagasaki 852-8523, Japan

## Abstract

Postnatally, scars occur as a consequence of cutaneous wound healing. Scarless wound healing is highly desired for patients who have undergone surgery or trauma, especially to exposed areas. Based on the properties of mesenchymal stem cells (MSCs) for tissue repair and immunomodulation, we investigated the potential of MSCs for scarless wound healing. MSCs were expanded from umbilical cord blood (UCB-MSCs) and Wharton’s jelly (WJ-MSCs) from healthy donors who underwent elective full-term pregnancy caesarean sections. UCB-MSCs expressed lower levels of the pre-inflammatory cytokines *IL1A* and *IL1B*, but higher levels of the extracellular matrix (ECM)-degradation enzymes *MMP1* and *PLAU* compared with WJ-MSCs, suggesting that UCB-MSCs were more likely to favor scarless wound healing. However, we failed to find significant benefits for stem cell therapy in improving wound healing and reducing collagen deposition following the direct injection of 1.0 × 10^5^ UCB-MSCs and WJ-MSCs into 5 mm full-thickness skin defect sites in nude mice. Interestingly, the implantation of UCB-MSCs tended to increase the expression of *MMP2* and *PLAU*, two proteases involved in degradation of the extracellular matrix in the wound tissues. Based on our data, UCB-MSCs are more likely to be a favorable potential stem cell source for scarless wound healing, although a better experimental model is required for confirmation.

Wound repair in skin is accomplished via complex processes^1^. Postnatally, wound repair in mammals commonly results in a non-functional mass of fibrotic tissue known as a scar^1^. Distinct from normal skin, scar tissue is characterized by a lack of epidermal appendages, changes in the structure of the basal cell layer of the epidermis, and alterations in extracellular matrix (ECM) structure and components^2^. The different color and texture of scar tissue in exposed areas such as the face or hands can also decrease quality of life. Some anti-scarring therapies, including the physical relaxation of tension in the wound margins and the administration of tranilast or recombinant human transforming growth factor β_3_, have been clinically tested^3^,^4^, but exhibited limited marginal effects. Therefore, scarless wound healing is highly desired for patients who have suffered surgery or trauma, especially to exposed areas.

Although the mechanisms are not completely understood, scarred wound healing is thought to be associated with the immune response, especially persistent inflammatory responses^5^. The rapid advancement in stem cell studies has clearly demonstrated that mesenchymal stem cells (MSCs) represent promising stem cell sources for the repair/regeneration of damaged tissues/organs. Beyond their application for repairing damaged tissues, MSCs have also been used for the treatment of autoimmune diseases and GVHD (graft versus host disease) due to their immunoregulatory properties^6^. To date, many clinical studies have demonstrated that MSCs can promote the healing of different wounds, including chronic skin ulcers induced by diabetic mellitus, radiation exposure, and ischemia^7^,^8^. Recently, the benefits of MSCs in a rabbit ear hypertrophic scar model were also reported^9^. Considering the immunoregulatory properties of MSCs, it is quite possible that MSCs favor scarless wound healing.

In this study, we investigated the potential of MSCs from umbilical cord blood (UCB-MSCs) and Wharton’s Jelly (WJ-MSCs), the most commonly used mesenchymal stem cell sources from fetuses^10^, for scarless wound healing. Furthermore, we attempted to elucidate the relevant mechanisms on stem cell-based therapy for scarless wound healing.

## Results

### Cell growth, morphological features, and phenotypic characterization of UCB-MSCs and WJ-MSCs

We expanded a sufficient number of WJ-MSCs from 8 of the 9 Warton’s jelly samples within 4 weeks; however, one sample failed to grow cells due to a technical error (Supplemental table 2). By contrast, only 2 of the 13 UCB samples finally expanded to over one million MSCs within 6 weeks (Supplemental table 2). To reduce individual variation due to differences in genetic and non-genetic backgrounds, we used UCB-MSCs and WJ-MSCs expanded from the same donor for the following experiments.

**Figure 1:**
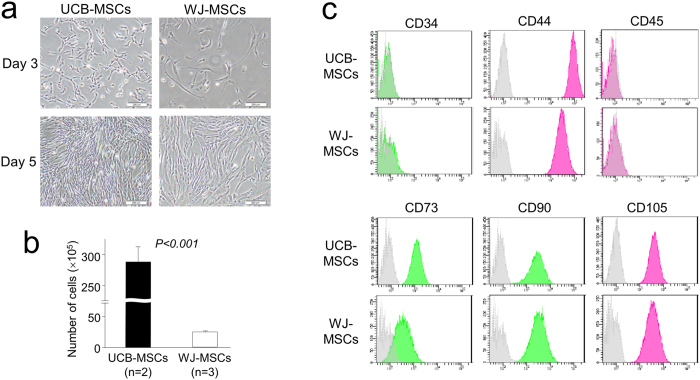
Morphological and phenotypical characterization of mesenchymal stem cells derived from umbilical cord blood (UCB-MSCs) and Warton’s jelly (WJ-MSCs). (**a**) Representative images of UCB-MSCs and WJ-MSCs from first passage cells demonstrating the fibroblast-like spindle shape after 3 and 5 days in culture. The UCB-MSCs were shown to be smaller in size and exhibited higher nuclear fluctuation (40×, scale bars 200 μm). (**b**) The cell growth of UCB-MSCs was significantly faster than WJ-MSCs. (**c**) Flow cytometry analysis expression profiles of the cell surface markers CD34, CD44, CD45, CD73, CD90, and CD105 in UCB-MSCs and WJ-MSCs. A representative histogram is presented. The colored lines and colored areas represent the expression of UCB-MSCs and WJ-MSCs, respectively. The gray areas represent the isotype negative control.

Although both of the UCB-MSCs and WJ-MSCs adopted fibroblast-like spindle shapes, the UCB-MSCs were smaller in size and featured higher nuclear fluctuation (Fig. 1a). The UCB-MSCs also grew much faster than WJ-MSCs (Fig. 1b).

Agreed well with previous studies^11^,^12^, UCB-MSCs and WJ-MSCs expressed the putative mesenchymal markers CD44, CD73, CD90 and CD105, but not the hematopoietic markers CD34 and CD45 (Fig. 1c). These results confirmed the phenotypic characterization of cells as mesenchymal stem cells.

### Differential expression of genes associated with wound healing and fibrosis between UCB-MSCs and WJ-MSCs

**Figure 2:**
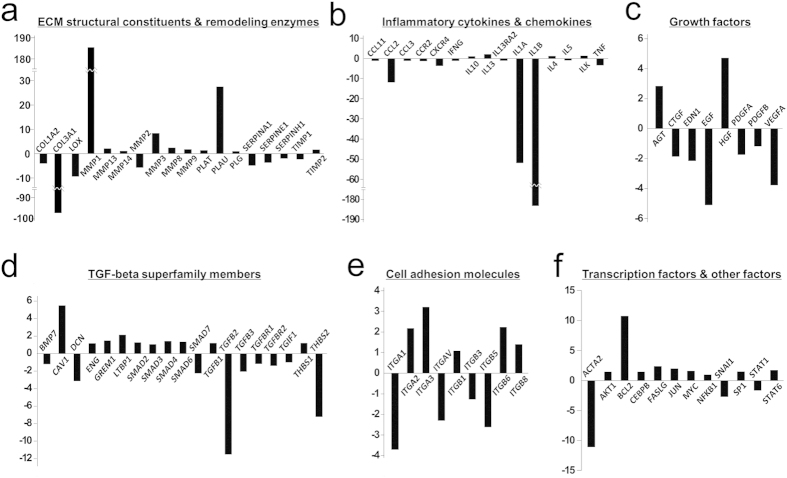
RT^2^ Profiler PCR array to detect the expression of genes associated with wound healing and fibrosis. We functionally categorized the genes into ECM structural constituents and remodeling enzymes (**a**), inflammatory cytokines and chemokines (**b**), growth factors (**c**), TGF-beta superfamily members (**d**), cell adhesion molecules (**e**), and transcription factors and other fibrosis factors (**f**). The data are presented as the means of fold change of expression in UCB-MSCs compared with WJ-MSCs from three separate experiments.

We extensively compared the expression of genes associated with wound healing and fibrosis in UCB-MSCs and WJ-MSCs using the RT^2^ Profiler PCR array. Interestingly, the expression levels of some genes were largely different between UCB-MSCs and WJ-MSCs even when the cells were expanded from the same donor (Fig. 2 and Supplemental table 3). For example, *MMP1* and *PLAU*, which are involved in the degradation of ECM^13^,^14^, were highly expressed in UCB-MSCs compared with WJ-MSCs (Fig. 2a). By contrast, the expression of *COL3A1*, a gene known to produce the component of type III collagen, was observed at much lower levels in UCB-MSCs than in WJ-MSCs (Fig. 2a). Furthermore, UCB-MSCs expressed much lower levels of *IL1A* and *IL1B* encoding the pro-inflammatory cytokines interleukin (IL)-1alpha and IL-1beta compared with WJ-MSCs (Fig. 2b), but expressed higher levels of *HGF*, an anti-fibrotic growth factor known to improve wound healing (Fig. 2c).

### Neither UCB-MSCs nor WJ-MSCs accelerated wound healing in nude mice

**Figure 3:**
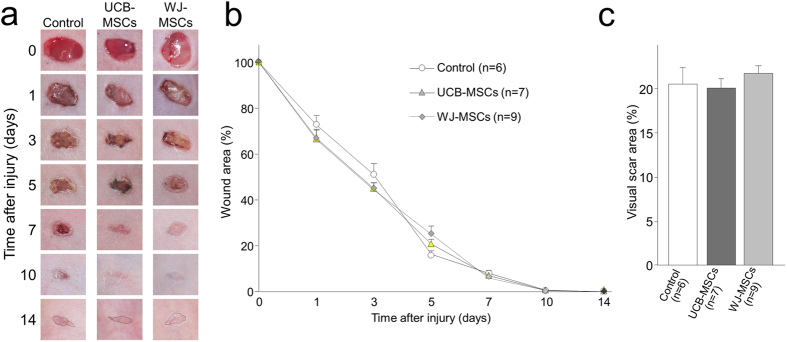
Wound healing of full-thickness skin defects (5 mm diameter) in nude mice. The wounds of mice were injected with mesenchymal stem cells derived from human umbilical cord blood (UCB group) and Warton’s Jelly (WJ group) or medium alone (Control group), and wound closure was recorded macroscopically after treatments. (**a**) Representative images of the gross appearance of excisional wounds over time after treatment. (**b**) Quantitative data concerning the proportion of the wound remaining open relative to the initial wound area at different time points after treatment. (**c**) Quantitative data on the visual scar area 14 days after treatment. The visual scar area was carefully circled on the images of wounds (dark dotted lines in Figure 3a). The percentage of the scar area was calculated relative to the initial wound area.

We directly injected UCB-MSCs and WJ-MSCs into the wound sites of 5 mm diameter full-thickness skin defects in nude mice, and then recorded the closure of the wounds over time. The wounds healed very well in all of the mice. Macroscopically, the wounds were almost closed at 7 days, and appeared as scar tissues 14 days after treatment (Fig. 3a). There was no obviously difference in the speed of wound closure among the groups during the 14 day follow-up period. Quantitative analysis showed that the measured wound area was slightly smaller in mice who received either UCB-MSCs or MJ-MSCs compared with the mice who received the control treatment at 1 and 3 days, but there was no significant difference among the groups (Fig. 3b). We also visually measured the scar area (dotted lines in the pictures of Fig. 3a) 14 days after treatment, but again there was no significant difference among the groups (Fig. 3c).

**Figure 4:**
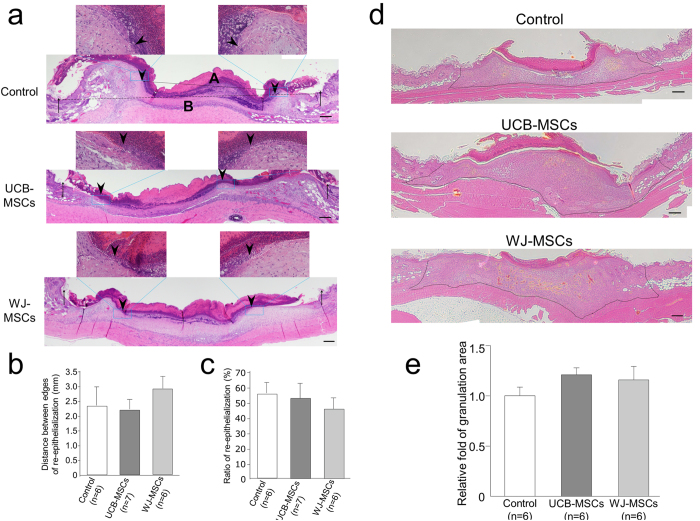
Histological analysis of the re-epithelialization and granulation of wounds 3 and 7 days after treatment. (**a**) Representative images of wound tissues with HE staining 3 days after treatment. The original wound margins (indicated with arrows) and the re-epithelialization edges (arrowheads) were carefully identified under the microscope. The distances between two wound margins (B; wound lengths) and between two re-epithelialization edges (A) were measured. The images of whole wound tissues were taken under a low power lens (40×, scale bars 200 μm). The inset high-power (200×) images indicate the epithelial tongues (surrounded by the dotted white lines) of the wounds. (**b**) Quantitative data on the distances between the re-epithelialization edges 3 days after treatment. (**c**) The ratio of re-epithelialization was measured by: [the wound length (B) minus the distances between the re-epithelialization edges (A)]/B × 100. (**d**) Representative images of wound tissues with HE staining 7 days after treatment. The granulation area was surrounded by the dotted black lines (40×, scale bars: 200 μm). (**e**) The area of granulated tissue was measured by Adobe Photoshop CS6 Extended software.

Because these wounds were almost closed within 7 days, we considered the wounds to be in the proliferation phase from 3 to 7 days post-treatment^15^. Therefore, we harvested wound tissues for histological analysis of the re-epithelialization 3 days post-treatment. We could easily see the re-epithelialized wound tongues at two wound sites from the images of HE-stained sections (Fig. 4a). We measured the width of the wound and the length of the re-epithelialization (the width of the wound minus the distance between re-epithelialized wound tongues); the ratio of re-epithelialization was calculated by the length of the re-epithelialization/the width of wound ×100. There was no significant difference in the re-epithelialization among the groups (Fig. 4b,c), suggesting that the implantation of UCB-MSCs and WJ-MSCs did not accelerate the re-epithelialization of full-thickness skin defect wounds in nude mice.

We also measured the amount of granulation in the wound tissues 7 days post-treatment using the HE-stained sections (Fig. 4d). Unexpectedly, the mice who received the UCB-MSC and WJ-MSC implantations produced an increased amount of granulated tissue compared with the control group of mice (1.21 ± 0.07 in the UCB group and 1.16 ± 0.13 in the WJ group *versus* 1.00 ± 0.01 in the Control group), although there was no significant difference among the groups (Fig. 4e).

### Neither UCB-MSCs nor WJ-MSCs contributed to scarless wound healing in nude mice

**Figure 5:**
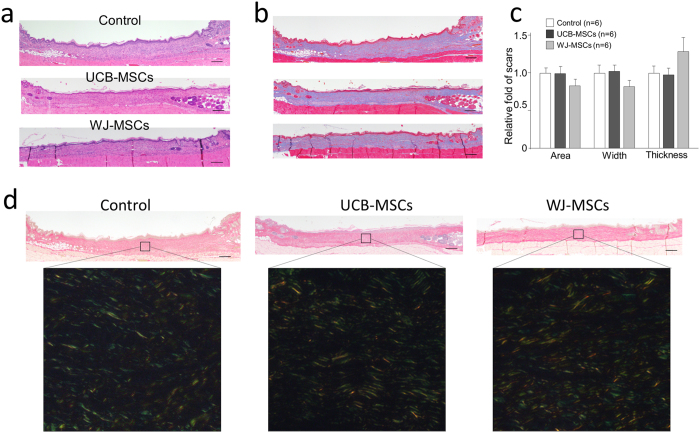
Histological analysis of scar formation of the healed wounds 14 days after treatment. (**a**) Representative images of wound tissues with HE staining (40×, scale bars 200 μm). (**b**) Representative images of wound tissues with Masson’s trichrome staining (40×, scale bars 200 μm); the scar tissues stained blue. (**c**) Quantitative data on the area, width, and thickness of scars based on the images of tissue sections with Masson’s trichrome staining. (**d**) Representative images of wound tissues with Picrosirius red staining for analysis of collagen fibers. The inserted high-magnification images in the lower panel were used to detect the alignment of type I collagen (yellow) and type III collagen (green) in the scar using polarized light microscopy.

We ended the follow-up 14 days after treatment because the scars were considered to be almost matured. We did not observe the regeneration of skin appendages in any of the mice at the endpoint of follow-up (Fig. 5a). To evaluate scarless wound healing, we measured collagen deposition by Masson trichrome staining (Fig. 5b). Semi-quantitative analysis showed that scar tissue with obvious collagen deposition (stained in blue) did not significantly differ between groups (Fig. 5c), although the scar tissue in the WJ-MSCs group exhibited a thicker, lower width, and smaller area compared with the other two groups. We performed Picrosirius red staining to detect type I and III collagen fibers (Fig. 5d). Although quantitative analysis was difficult, positive staining for type I and III collagen fibers was observed to be similar among the groups (Fig. 5d). These findings suggested that UCB-MSCs and WJ-MSCs did not significantly contribute to scarless wound healing in nude mice.

### The implantation of UCB-MSCs and WJ-MSCs into the wounds of nude mice tended to increase collagen synthesis and inflammatory cytokine production

**Figure 6:**
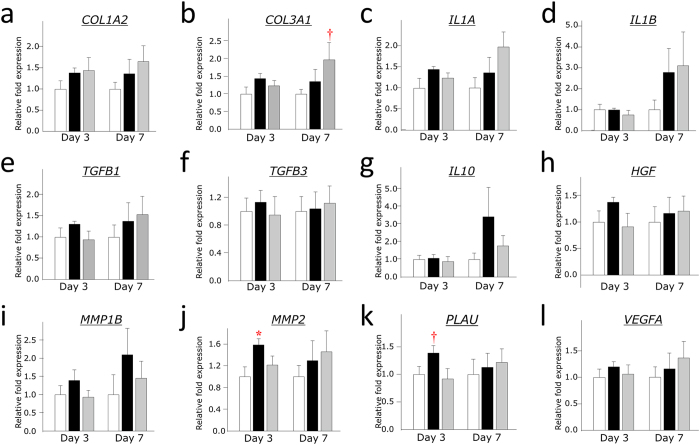
RT-PCR analysis of the expression of important genes associated with wound healing in wound tissues of nude mice. Tissue samples were collected 3 days (n = 6 in each group) and 7 days (n = 6 in the Control group and n = 7 in the UCB-MSC and WJ-MSC groups) after treatment. The quantitative data from the RT-PCR was normalized by the expression of 18S ribosomal RNA. 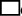
Control group, 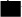
UCB-MSC group, 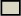
WJ-MSC group. *p < 0.05 *vs.* Control group. †p < 0.10 *vs.* Control group.

We also investigated angiogenesis, the recruitment of macrophages, and the expression of several inflammatory cytokines and growth factors that are known to be closely associated with the wound healing process in the wound tissues 3 and 7 days after treatment (Fig. 6). The results were in agreement with the histological findings. WJ-MSCs implantation tended to enhance the expression of *COL3A1* 7 days after treatment (p = 0.078 *vs.* Control group, Fig. 6b). Although the expression of some inflammatory cytokines, such as *IL1A* and *IL1B,* was increased in the wound tissues of mice treated with UCB-MSCs and WJ-MSCs (Fig. 6c,d), but was not significant different among the groups. These data suggested that the xenograft of human UCB-MSCs and WJ-MSCs into the wounds of nude mice might enhance collagen synthesis and the inflammatory response. Interestingly, the implantation with UCB-MSCs, but not WJ-MSCs increased some genes associated with ECM remodeling, including *MMP2* (p = 0.019 *vs.* Control group, Fig. 6j) and *PLAU* (p = 0.080 *vs.* Control group, Fig. 6k), 3 days after treatment. Although the expression of the anti-inflammatory cytokine *IL10* and anti-fibrotic factor *HGF* was also increased by the implantation with UCB-MSCs (Fig. 6g,h), there was not significant different among the groups, due to the mall sample size and the large individual difference of samples.

**Figure 7:**
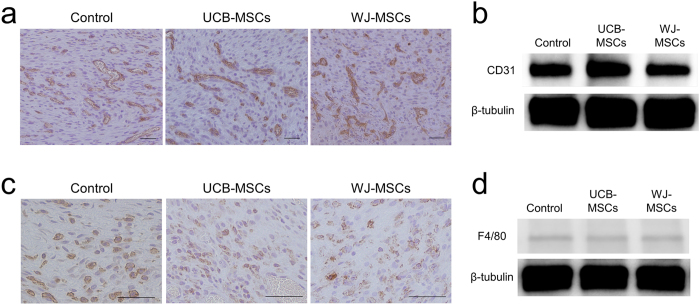
Angiogenesis and the infiltration of macrophages into wound tissues of nude mice 7 days after treatment. (**a**) Representative images of immunohistochemistry staining with the endothelial marker CD31 (400×, scale bars 50 μm). The density of microvessels that positively stained for CD31 was observed to be similar in the wound tissues among groups. (**b**) Western immunoblot analysis of CD31 expression in wound tissues. (**c**) Representative images of immunostaining with F4/80, a marker for macrophages (400×, scale bars 50 μm). (**d**) Western immunoblot analysis of F4/80 expression.

We did not observe obvious differences in the expression of the angiogenesis marker CD31 among the groups by IHC staining or western blotting analysis (Fig. 7a,b). Similarly, there was no obvious difference in macrophage infiltration into the wound tissues among the groups (Fig. 7c,d).

## Discussion

Scarless wound healing is highly desired for patients who have suffered surgery or trauma, especially to exposed areas. We selected UCB-MSCs and WJ-MSCs as the candidate sources of stem cells to test for scarless wound healing because of the following reasons: 1) MSCs of different origins have been demonstrated to promote wound healing and have been clinically applied for the treatment of skin ulcers^16^,^17^; 2) MSCs have immunomodulation properties^12^,^18^, indicating their potency for anti-fibrotic/scarring therapy; 3) some pediatric patients require a surgical procedure due to congenital diseases, and a sufficient amount of umbilical cord blood and Wharton’s jelly tissue are easily obtained without the need for invasive procedures; and 4) the isolation and *ex vivo* expansion of MSCs from umbilical cord blood and Wharton’s jelly has been technically accomplished^10^, and these expanded UCB-MSCs and WJ-MSCs could be stocked for future applications.

Similar to a previous report^19^, we easily expanded a sufficient number of cells from Wharton’s jelly; moreover, these expanded cells were characterized as MSCs. However, although we collected a sufficient volume of umbilical cord blood (33–85 ml/donor) and used a protocol similar to a previously described method to expand MSCs^12^,^18^, we failed to grow a sufficient amount of MSCs from the majority of the donor samples. The reason for this technical failure is unclear, but another study reported the same problem (<10% success rate)^12^. Although MSC allografts have been clinically applied for immune diseases^6^, an autograft of stem cell therapy is naturally considered to be a superior option for scarless wound healing. Therefore, methodological modifications are needed to improve the successful *ex vivo* expansion rate of UCB-MSCs.

MSCs are known to exist as a heterogeneous population. Recently, an attempt to biologically characterize bone marrow-derived MSCs with the use of various biophysical markers was reported^20^. These MSCs were shown to possess a small size with high nuclear fluctuation, thereby predicting the “stemness” and enhanced therapeutic capacity. Interestingly, the UCB-MSCs in this study were observed to be smaller in size with higher nuclear fluctuation compared with WJ-MSCs. Further studies are required to understand the biological significance of the different morphological features between WJ-MSCs and UCB-MSCs, and also to determine whether UCB-MSCs possess better properties for tissue regeneration/repair.

The most interesting findings of this study were the differential expression of genes associated with fibrosis and wound healing between UCB-MSCs and WJ-MSCs. Our data showed that UCB-MSCs expressed higher levels of ECM remodeling enzymes, but lower levels of collagens. Furthermore, WJ-MSCs expressed higher levels of the pro-inflammatory cytokines IL-1alpha and IL-1beta^21^. Moreover, the anti-fibrotic wound healing factor HGF was also highly expressed in UCB-MSCs. These gene expression profiles strongly suggest that UCB-MSCs may represent a potential stem cell source for scarless wound healing.

Previous studies clearly showed the benefits of UCB-MSCs and WJ-MSCs for wound healing^16^,^17^. However, we failed to confirm the benefit of UCB-MSCs and WJ-MSCs in wound healing using the well-established full-thickness skin defect model in nude mice by histological analyses. Neither UCB-MSCs nor WJ-MSCs favored scarless wound healing in our experimental animal model. By contrast, our data indicated that the injection of UCB-MSCs and WJ-MSCs into dermal defect sites in nude mice increased the expression of TGF-beta1 and collagens rather than attenuate scar formation. The expression of *IL1A* and *IL1B*, two inflammatory cytokines that are known to stimulate the proliferation of skin fibroblasts and promote their differentiation into myofibroblasts^22^, was also increased in the wound tissues 7 days after implantation with UCB-MSCs and WJ-MSCs.

Scars are known to form as a result of a sequence of biological responses in wounds, such as angiogenesis, inflammation, granulation and ECM remodeling. Therefore, the negative data from our *in vivo* investigations could have several explanations. The first is the issue of the choice of the experimental animal model. A persistent inflammatory response is considered to play key roles in scarred wound healing, but we used immunodeficient nude mice for the experiment. Cutaneous wounds in nude mice have been reported to heal with less scarring and lower levels of collagen deposition^23^. In this study, the 5 mm full-thickness skin defect healed very fast and looked almost like normal skin within 14 days, even in the mice who received the control treatment. Therefore, whether nude mice should be used to test scarless wound healing remains an open question.

Second, it is possible that the number of administered cells was too small to have a sufficient therapeutic effect. Previous studies reported different results when UCB-MSCs and WJ-MSCs were used for wound healing by direct injection of >3.0 × 10^5^ cells into a 5 mm full-thickness skin defect^16^,^17^; by contrast, we injected only 1.0 × 10^5^ cells in this study. The survival rate of cells has been reported to be very poor after injection into the wound site^24^, and the improvement of cell retention using scaffolds has been demonstrated to increase the effect of stem cell therapy^25^. Further experiments involving the injection of more cells are needed to investigate the beneficial effect of UCB-MSCs and WJ-MSCs for scarless wound healing.

Finally, the immunomodulatory properties of UCB-MSCs and WJ-MSCs may not be easily observed in nude mice due to the deficiency of T cells^23^. However, the production of the inflammatory cytokines IL-1alpha and IL-1beta were enhanced in the wound tissues that received the implantation with UCB-MSCs and WJ-MSCs. This result suggested that the xenograft of human MSCs into nude mice might also increase immune responses, thereby negating the benefit of UCB-MSCs and WJ-MSCs for scarless wound healing.

Although the overall data of our *in vivo* investigations were considered to be negative, some parameters, such as the expressions of anti-fibrotic growth factor HGF and anti-inflammatory cytokine IL-10, were increased in the wound tissues at 3 or 7 days after implantation with UCB-MSCs, but not WJ-MSCs. Similarly, only the implantation with UCB-MSCs enhanced the expression of ECM-degradation enzymes (i.e., *MMP2* and *PLAU*) in the wounds. Because the production of cytokines and growth factors has been demonstrated to play critical roles in the immunomodulatory property of MSCs^6^, these data provide indirect evidence that UCB-MSCs may favor scarless wound healing through paracrine mechanisms. However, further studies are required to confirm our speculation.

In summary, we examined the potency of UCB-MSCs and WJ-MSCs for scarless wound healing. Gene expression profiles showed that the UCB-MSCs expressed much higher levels of *MMP1* and *PLAU*, but lower levels of the inflammatory cytokines of *IL1A* and *IL1B*. Although we failed to demonstrate the benefit of UCB-MSCs and WJ-MSCs for scarless wound healing in a full-thickness skin defect model of nude mice, the implantation of UCB-MSCs increased the expression of several ECM-degradation enzymes in the wound tissues during the proliferation phase. Thus, UCB-MSCs are more likely to be a potential stem cell source that is favorable for scarless wound healing.

## Materials and methods

### Ethics

This study was approved by the Nagasaki University Graduate School of Biomedical Sciences clinical research ethics committee (12053003). Placental and umbilical cord tissues were collected from healthy donors who underwent elective caesarean sections after full-term pregnancy; all donors provided informed consent.

All animal experiments were approved by the Ethics Review Committee for animal experimentation (1206070993-2) at Nagasaki University (Nagasaki, Japan). The experiments were performed in accordance with the institutional and national guidelines.

### *Ex vivo* expansion of UCB-MSCs

Umbilical cord blood was collected from delivered placentas using sterile 18G needles and 50 ml syringes contained 1000 units of heparin. Collected blood samples were stored on ice and diluted with Dulbecco’s phosphate-buffer saline (D-PBS) containing 2 mM EDTA. Mononuclear cells were isolated by density gradient centrifugation using Ficoll-Hypaque-Plus solution (GE Healthcare). Freshly isolated mononuclear cells were suspended in Dulbecco’s modified Eagle’s medium-low glucose (DMEM-LG) (Wako) containing 10% fetal bovine serum (Hyclone Laboratories, Inc.) and 1% penicillin/streptomycin (Life Technologies), and then seeded into T75- or T25-cell culture flasks at a density of 8.0–10 × 10^5^ cells/cm^2^. Cells were incubated at 37 °C in 5% CO_2_, and the medium was changed every 3–4 days. We continued the cultures for approximately 1 week after fibroblast-like cells appeared on the bottom of the flasks. Then, these fibroblast-like cells were collected using 0.25% trypsin-EDTA and re-plated for further *ex vivo* expansion. The 2^nd^ or 3^rd^ passage cells were used for experiments, excluding the cell growth assay.

### *Ex vivo* expansion of WJ-MSCs

Umbilical cord tissue was collected and stored in Hank’s balanced salt solution (Life Technologies) at 4 °C. After removing the umbilical artery and veins, the Wharton’s jelly tissue was cut into small pieces (1–2 mm) and placed into 6 or 10 cm culture dishes coated with 10 μg/ml human fibronectin. A small amount of DMEM-LG media containing 10% FBS and penicillin/streptomycin was added to the dishes, and the cells were incubated in 5% CO_2_ at 37 °C. After overnight incubation, more media was added to the dishes. Fibroblast-like cells grew out from the tissues after approximately 5 days of incubation with medium changes every 3–4 days. The cells were harvested using 0.25% trypsin-EDTA and re-plated for further *ex vivo* expansion. We used 2^nd^ passage cells for experiments, excluding the cell growth assay.

### Cell growth assay

First passage UCB-MSCs and WJ-MSCs were seeded into 6-well plates at a density of 3.0 × 10^3^ cells/cm^2^ and cultured for 1 week. The cells were collected as a single cell suspension, and the total cell numbers were counted with the NucleoCounter^®^ NC-100^TM^.

### Flow cytometry

To characterize the UCB-MSCs and WJ-MSCs, 2^nd^ passage cells were trypsinized to generate a single cell suspension, and then stained with mouse monoclonal antibodies against CD34-FITC (4H11), CD44-PE (IM7), CD45-PE (HI30), CD73-FITC (AD2), CD90-FITC (eBio5E10), and CD105-PE (SN6) (eBioscience). The respective isotype controls were used as negative controls. After washing twice with PBS, quantitative flow cytometry analysis was performed using an LSRFortessa^TM^ instrument (Becton Dickinson). We analyzed the acquired data using the Cell Quest software (Becton Dickinson).

### RT^2^ Profiler PCR array

Total RNA was isolated from UCB-MSCs and WJ-MSCs using the RNeasy Mini Kit (Qiagen). After the generation of cDNA using the RT^2^ First Strand Kit (SABiosciences), the RT^2^ Profiler PCR array for human fibrosis was performed according to the manufacturer’s instructions (SABiosciences). A total of 84 key genes involved in tissue remodeling during the repair and healing of wounds were included in the array. Three separate experiments were done and data was analyzed by a web-based analysis program (SABiosciences, http://pcrdataanalysis.sabiosciences.com/pcr/arrayanalysis.php).

### Mouse wound healing model and cell implantation

Six-week-old BALB/cSlc (nu-nu) mice were purchased from Japan SLC. Four full-thickness skin defects were created in the dorsal skin using 5 mm diameter biopsy punches (Kai Industries). Then, the mice were randomly divided into 3 groups and received subcutaneous injections at 4 points around each wound: a total number of 1.0 × 10^5^ UCB-MSCs in 50 μL medium (UCB-MSCs group), 1.0 × 10^5^ WJ-MSCs (WJ-MSCs group), or 50 μL medium only (Control group). The wounds were recorded using a digital camera (Lumix DMC-GF3, Panasonic) 0, 1, 3, 5, 7, 10 and 14 days after treatment, and the wound areas were measured using Photoshop CS6 Extended software (Adobe systems). Wound tissues were harvested with 1 mm normal skin margins at different time points for the following analyses.

### Real-time quantitative polymerase chain reaction (RT-PCR)

To measure the mRNA expression levels of several genes associated with wound healing, we harvested skin wound tissues for RT-PCR analysis 3 or 7 days after treatment. Briefly, wound tissues were homogenized using Multi-beads shocker^®^ (Yasui Kikai), and RNA purification were performed using ISOGEN2 (Nippon Gene) according to the manufacturer’s protocol. The ReverTra Ace^®^ q-PRC RT kit with genomic DNA remover (TOYOBO) was used to prepare the cDNA from the purified total RNA. RT-PCR was performed using a THUNDERBIRD SYBR qPCR mix (TOYOBO) and CFX96 Touch real-time PCR detection system (Bio Rad). The primer sequences used to quantify gene expression levels are listed in Supplemental table 1.

### Histological evaluation

For histological analysis, mice were killed 3, 7 and 14 days after treatment. The harvested wound tissues were fixed in 4% paraformaldehyde for paraffin embedding. Sections (6 μm thick) were used for various staining techniques: hematoxylin-eosin (HE) staining, Masson’s trichrome staining, Picrosirius red staining^26^, and immunohistochemistry (IHC) staining for CD31 (ab28364, Abcam) and F4/80 (CI:A3-1, Abcam). For IHC, sections were deparaffinized, incubated for 20 min with 3% hydrogen peroxide to block endogenous peroxidase activity, incubated with Blocking One Histo (Nacalai Tesque) at room temperature, and then incubated with primary antibodies at 4 °C overnight. All sections were visualized using Histofine Simplestaining mouseMAX-PO (rabbit or rat) (Nichirei Biosciences, Inc.) and 3,3′-Diaminobenzidine tablets (Sigma-Aldrich). Stained sections were viewed by microscopy (Olympus IX83, Olympus), and digital images were acquired with a DP80 camera using the cellSens software (Olympus). Picrosirius red stained sections were observed using a polarized microscope. HE-stained sections were used to evaluate the re-epithelialization and granulation of wounds. Images were optimized globally and assembled into figures using Adobe Photoshop.

### Western blots

To measure the CD31 and F4/80 protein levels, mice were sacrificed and wound tissues were harvested 7 days after treatment. Briefly, the harvested wound tissues were homogenized using Multi-beads shocker^®^ and added to the T-PER Reagent (Thermo Fisher Scientific) consisting of proteinase and dephosphorylation inhibitors (Thermo Fisher Scientific). Total protein lysates were obtained after the elimination of debris by filtering with 0.22 μm filters (Millipore). Total protein (10 μg) was separated using a mini-protean TGX Stain-Free^TM^ gel (BioRad), and then transferred to PVDF membranes using the Trans-Blot^®^ Turbo^TM^ transfer system (BioRad). After blocking with the PVDF Blocking Reagent Can Get Signal^®^ (TOYOBO) for 1 hour at room temperature, the membranes were incubated with primary antibodies against CD31, F4/80, or tubulin (Sigma-Aldrich) at 4 °C overnight, followed by the appropriate horseradish peroxidase-conjugated secondary antibodies at room temperature for 1 hour. Expression was visualized by chemiluminescence with a digital luminescent image analyzer (LAS-4000, GE Healthcare).

### Statistical analysis

Significant differences were determined using Student’s *t*-test, with p < 0.05 considered to be statistically significant. All data were presented as the means ± SEMs.

## Additional Information

**How to cite this article**: Doi, H. *et al.* Potency of umbilical cord blood- and Wharton’s jelly-derived mesenchymal stem cells for scarless wound healing. *Sci. Rep.* **6**, 18844; doi: 10.1038/srep18844 (2016).

## Supplementary Material

Supplementary Information

## References

[b1] Gurtner G. C., Werner S., Barrandon Y. & Longaker M. T. Wound repair and regeneration. Nature 453, 314–321, 10.1038/nature07039 (2008). 18480812

[b2] Martin P. Wound healing–aiming for perfect skin regeneration. Science (New York, N.Y.) 276, 75–81 (1997). 10.1126/science.276.5309.75 9082989

[b3] Shin D. & Minn K. W. The effect of myofibroblast on contracture of hypertrophic scar. Plastic and reconstructive surgery 113, 633–640, 10.1097/01.prs.0000101530.33096.5b (2004). 14758226

[b4] Ferguson M. W. *et al.* Prophylactic administration of avotermin for improvement of skin scarring: three double-blind, placebo-controlled, phase I/II studies. Lancet 373, 1264–1274, 10.1016/s0140-6736(09)60322-6 (2009). 19362676

[b5] Larson B. J., Longaker M. T. & Lorenz H. P. Scarless fetal wound healing: a basic science review. Plastic and reconstructive surgery 126, 1172–1180, 10.1097/PRS.0b013e3181eae781 (2010). 20885241 PMC4229131

[b6] Le Blanc K. & Mougiakakos D. Multipotent mesenchymal stromal cells and the innate immune system. Nature reviews. Immunology 12, 383–396, 10.1038/nri3209 (2012). 22531326

[b7] Bura A. *et al.* Phase I trial: the use of autologous cultured adipose-derived stroma/stem cells to treat patients with non-revascularizable critical limb ischemia. Cytotherapy 16, 245–257, 10.1016/j.jcyt.2013.11.011 (2014). 24438903

[b8] Yoshikawa T. *et al.* Wound therapy by marrow mesenchymal cell transplantation. Plastic and reconstructive surgery 121, 860–877, 10.1097/01.prs.0000299922.96006.24 (2008). 18317135

[b9] Liu S. *et al.* Mesenchymal stem cells prevent hypertrophic scar formation via inflammatory regulation when undergoing apoptosis. The Journal of investigative dermatology 134, 2648–2657, 10.1038/jid.2014.169 (2014). 24714203

[b10] Pelosi E., Castelli G. & Testa U. Human umbilical cord is a unique and safe source of various types of stem cells suitable for treatment of hematological diseases and for regenerative medicine. Blood cells, molecules & diseases 49, 20–28, 10.1016/j.bcmd.2012.02.007 (2012). 22446302

[b11] Kern S., Eichler H., Stoeve J., Kluter H. & Bieback K. Comparative analysis of mesenchymal stem cells from bone marrow, umbilical cord blood, or adipose tissue. Stem cells 24, 1294–1301, 10.1634/stemcells.2005-0342 (2006). 16410387

[b12] Manca M. F. *et al.* Characterization of mesenchymal stromal cells derived from full-term umbilical cord blood. Cytotherapy 10, 54–68, 10.1080/14653240701732763 (2008). 18202975

[b13] Shin M. K. *et al.* The effects of platelet-rich clot releasate on the expression of MMP-1 and type I collagen in human adult dermal fibroblasts: PRP is a stronger MMP-1 stimulator. Molecular biology reports 41, 3–8, 10.1007/s11033-013-2718-9 (2014). 24293148

[b14] Lund L. R. *et al.* Plasminogen activation independent of uPA and tPA maintains wound healing in gene-deficient mice. The EMBO journal 25, 2686–2697, 10.1038/sj.emboj.7601173 (2006). 16763560 PMC1500865

[b15] Stramer B. M., Mori R. & Martin P. The inflammation-fibrosis link? A Jekyll and Hyde role for blood cells during wound repair. The Journal of investigative dermatology 127, 1009–1017, 10.1038/sj.jid.5700811 (2007). 17435786

[b16] Luo G. *et al.* Promotion of cutaneous wound healing by local application of mesenchymal stem cells derived from human umbilical cord blood. Wound repair and regeneration : official publication of the Wound Healing Society [and] the European Tissue Repair Society 18, 506–513, 10.1111/j.1524-475X.2010.00616.x (2010). 20840520

[b17] Fong C. Y. *et al.* Human Wharton’s jelly stem cells and its conditioned medium enhance healing of excisional and diabetic wounds. Journal of cellular biochemistry 115, 290–302, 10.1002/jcb.24661 (2014). 24038311

[b18] Cutler A. J., Limbani V., Girdlestone J. & Navarrete C. V. Umbilical cord-derived mesenchymal stromal cells modulate monocyte function to suppress T cell proliferation. Journal of immunology 185, 6617–6623, 10.4049/jimmunol.1002239 (2010). 20980628

[b19] Cardoso T. C. *et al.* Isolation and characterization of Wharton’s jelly-derived multipotent mesenchymal stromal cells obtained from bovine umbilical cord and maintained in a defined serum-free three-dimensional system. BMC biotechnology 12, 18, 10.1186/1472-6750-12-18 (2012). 22559872 PMC3443425

[b20] Lee W. C. *et al.* Multivariate biophysical markers predictive of mesenchymal stromal cell multipotency. Proceedings of the National Academy of Sciences of the United States of America 111, E4409–4418, 10.1073/pnas.1402306111 (2014). 25298531 PMC4210311

[b21] Peranteau W. H. *et al.* IL-10 overexpression decreases inflammatory mediators and promotes regenerative healing in an adult model of scar formation. The Journal of investigative dermatology 128, 1852–1860, 10.1038/sj.jid.5701232 (2008). 18200061

[b22] Shephard P. *et al.* Myofibroblast differentiation is induced in keratinocyte-fibroblast co-cultures and is antagonistically regulated by endogenous transforming growth factor-beta and interleukin-1. Am J Pathol 164, 2055–2066 (2004). 15161640 10.1016/s0002-9440(10)63764-9 PMC1615767

[b23] Gawronska-Kozak B., Bogacki M., Rim J. S., Monroe W. T. & Manuel J. A. Scarless skin repair in immunodeficient mice. Wound repair and regeneration : official publication of the Wound Healing Society [and] the European Tissue Repair Society 14, 265–276, 10.1111/j.1743-6109.2006.00121.x (2006). 16808805

[b24] Wu Y. *et al.* Bone marrow-derived mesenchymal stem cell attenuates skin fibrosis development in mice. International wound journal 11, 701–710, 10.1111/iwj.12034 (2014). 23409729 PMC7950597

[b25] Sabapathy V., Sundaram B., V, M. S., Mankuzhy P. & Kumar S. Human Wharton’s Jelly Mesenchymal Stem Cells plasticity augments scar-free skin wound healing with hair growth. PloS one 9, e93726, 10.1371/journal.pone.0093726 (2014). 24736473 PMC3988008

[b26] Junqueira L. C., Bignolas G. & Brentani R. R. Picrosirius staining plus polarization microscopy, a specific method for collagen detection in tissue sections. The Histochemical journal 11, 447–455 (1979) 91593 10.1007/BF01002772

