# Supplementary material for: Potency of umbilical cord blood- and Wharton’s jelly-derived mesenchymal stem cells for scarless wound healing
Source: Sci Rep. 2016 Jan 5;6:18844. doi: 10.1038/srep18844 (PMC4700425; doi:10.1038/srep18844)
Supplement: Supplementary Information [file srep18844-s1.pdf]

Potency of umbilical cord blood- and Wharton's jelly-derived mesenchymal stem cells for scarless wound healing

Hanako Doi, Yuriko Kitajima, Lan Luo, Chan Yan, Seiko Tateishi, Yusuke Ono, Yoshishige Urata, Shinji Goto, Ryoichi Mori, Hideaki Masuzaki, Isao Shimokawa, Akiyoshi Hirano, Tao-Sheng Li

Supplemental table 1. The primer sequences used to quantify gene expression levels.

gene	Sequence (5'->3')	
	Forward primer	Reverse primer
HGF	GCCCTATTTCCCGTTGTGAAGG	CCCGCAGTTGTTTTGTTTTGGC
MMP2	CTGGTCGCAGTGATGGCTTC	GTCCATCTGCATTGCCACCC
PLAU	AACAGATTCCTGCAAGGGCGA	CCCAGCTCACAATCCCACTCA
TGFB1	CGTCACTGGAGTTGTACGGC	TTGGGGCTGATCCCGTTGA
IL-1A	GTCGGCAAAGAAATCAAGATGGC	TCAGAGAGAGATGGTCAATGGCA
IL-1B	TGCCACCTTTTGACAGTGATGAG	TGTTGATGTGCTGCTGCGAG
IL-10	AGGCGCTGTCATCGATTTCTC	CATGGCCTTGTAGACACCTTGG
VEGFA	CTCCACCATGCCAAGTGGTC	ACCAGGGTCTCAATCGGACG
18S ribosomal RNA	TTCTGGCCAACGGTCTAGACAAC	CCAGTGGTCTTGGTGTGCTGA
COL1A2	GGATACGCGGACTCTGTTGC	CCTTTCGTACTGATCCCGATTGC
COL3A1	TGGACTGAAGGGTGAAAATGGTCT	CCGAGCACCATCATTGCCTC
TGFB3	CGAGTGGCTGTTGAGGAGAG	TCATCTTCATTGTCCACTCCTTTGA
MMP1B	AGCTTCCTGCCAGAACCTTTGA	AGCCCATACTTTGCTGCCTTTG

Supplemental table 2. Information about the isolation and ex vivo expansion of mesenchymal stem cells from umbilical cord blood (UCB-MSCs) and Wharton's jelly (WJ-MSCs)

Donor number	WJ-MSCs	UCB-MSCs
**#1**	○	×
**#2**	○	×
**#3**	○	×
**#4**	○	×
**#5**	○	○
**#6**	△	×
**#7**	○	-
**#8**	○	×
**#9**	-	×
**#10**	-	×
**#11**	-	×
**#12**	-	○
**#13**	-	×
**#14**	-	×

○: successfully expanded a sufficient number of cells (>1,000,000)

×: failed to expand a sufficient number of cells.

△: the most of tissues (explants) failed to attach to dishes due to technical error.

−: no procedure was performed.

Supplemental table 3. Results of RT² Profiler PCR array. The fold regulation represents the expression levels in umbilical cord blood-derived mesenchymal stem cells (UCB-MSCs) when compared to Wharton's jelly-derived mesenchymal stem cells (WJ-MSCs). The data are presented as the means from three separate experiments. Bolded p-value shows significant difference (P<0.05).

**Inflammatory cytokines & chemokines**		
Gene symbol	Fold Regulation	p-value
CCL11	-1.19	0.197
CCL2	-11.83	**0.009**
CCL3	-1.19	0.197
CCR2	-1.31	**0.007**
CXCR4	-3.80	0.168
IFNG	-1.19	0.197
IL10	1.01	0.859
IL13	2.12	0.239
IL13RA2	-1.11	0.653
IL1A	-52.02	**0.005**
IL1B	-183.26	**0.026**
IL4	1.15	0.551
IL5	-1.06	0.974
ILK	1.27	0.448
TNF	-3.48	0.097
**TGF-beta superfamily members**		
Gene symbol	Fold Regulation	p-value
BMP7	-1.19	0.197
CAV1	5.49	0.102
DCN	-3.16	**0.026**
ENG	1.18	0.759
GREM1	1.48	0.191
LTBP1	2.13	**0.007**
SMAD2	1.26	0.099
SMAD3	1.05	0.836
SMAD4	1.42	**0.009**
SMAD6	1.34	0.400
SMAD7	-2.25	0.062
TGFB1	1.20	0.626
TGFB2	-11.51	**0.048**
TGFB3	-2.05	0.210
TGFBR1	-1.17	0.485
TGFBR2	-1.40	0.311
TGIF1	-1.01	0.751
THBS1	1.19	0.749
THBS2	-7.23	**0.029**

**ECM structural constituents & remodeling enzyme**		
Gene symbol	Fold Regulation	p-value
COL1A2	-4.01	**0.000**
COL3A1	-97.53	**0.003**
LOX	-9.37	**0.019**
MMP1	184.68	**0.000**
MMP13	2.07	0.262
MMP14	1.05	0.683
MMP2	-5.73	**0.024**
MMP3	8.37	0.237
MMP8	2.37	0.214
MMP9	1.76	0.301
PLAT	1.29	0.534
PLAU	27.39	**0.007**
PLG	1.00	0.819
SERPINA1	-4.88	**0.013**
SERPINE1	-3.68	0.106
SERPINH1	-1.98	**0.001**
TIMP1	-2.41	0.056
TIMP2	1.64	0.061
TIMP3	-8.06	0.063
TIMP4	2.17	0.060
**Growth factors**		
Gene symbol	Fold Regulation	p-value
AGT	2.83	0.381
CTGF	-1.88	0.103
EDN1	-2.16	0.155
EGF	-5.11	**0.013**
HGF	4.70	**0.013**
PDGFA	-1.74	**0.022**
PDGFB	-1.19	0.197
VEGFA	-3.79	0.259

**Cell adhesion molecules**		
Gene symbol	Fold Regulation	p-value
ITGA1	-3.71	0.117
ITGA2	2.18	0.106
ITGA3	3.21	**0.031**
ITGAV	-2.30	**0.008**
ITGB1	1.07	0.787
ITGB3	-1.27	0.158
ITGB5	-2.62	**0.016**
ITGB6	2.24	0.381
ITGB8	1.39	0.506
**Transcription factors**		
Gene symbol	Fold Regulation	p-value
CEBPB	1.47	0.368
JUN	2.02	0.275
MYC	1.61	0.159
NFKB1	1.01	0.890
SP1	1.50	**0.048**
STAT1	-1.66	0.124
STAT6	1.75	0.294
**Other fibrosis factors**		
Gene symbol	Fold Regulation	p-value
ACTA2	-11.09	**0.022**
AKT1	1.47	0.244
BCL2	10.74	0.053
FASLG	2.42	0.175
SNAI1	-2.68	0.080

Supplementary Figure 1.

Original images of blots for Figure 7-B and D. Squared frame with redline indicates the cropped area.

CD31

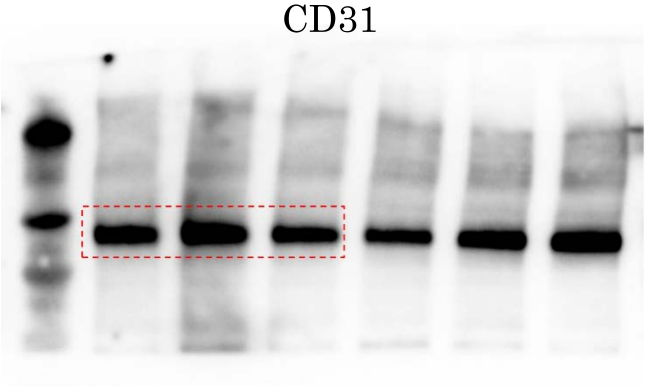

F4/80

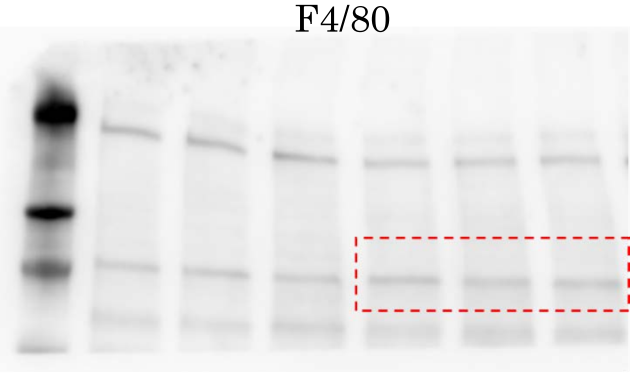

Tubulin

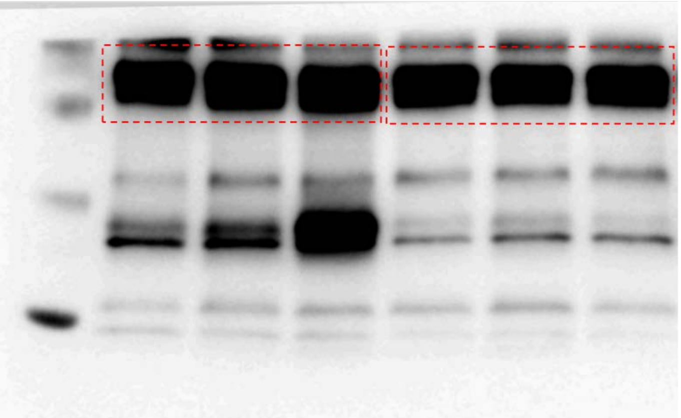

Right square frame indicates internal control of CD31
Left square frame indicates internal control of F4/80
